# Effect of zoledronic acid on the doxycycline-induced decrease in tumour burden in a bone metastasis model of human breast cancer

**DOI:** 10.1038/sj.bjc.6603740

**Published:** 2007-04-17

**Authors:** W C M Duivenvoorden, S Vukmirović-Popović, M Kalina, E Seidlitz, G Singh

**Affiliations:** 1 Juravinski Cancer Centre, 699 Concession Street, Hamilton, Ontario, Canada L8V 5C2; 2 Department of Pathology and Molecular Medicine, McMaster University, 1280 Main Street W, Hamilton, Ontario, Canada L8N 3Z5

**Keywords:** bone metastasis, breast cancer, tetracyclines, bisphosphonates

## Abstract

Bone is one of the most frequent sites for metastasis in breast cancer patients often resulting in significant clinical morbidity and mortality. Bisphosphonates are currently the standard of care for breast cancer patients with bone metastasis. We have shown previously that doxycycline, a member of the tetracycline family of antibiotics, reduces total tumour burden in an experimental bone metastasis mouse model of human breast cancer. In this study, we combined doxycycline treatment together with zoledronic acid, the most potent bisphosphonate. Drug administration started 3 days before the injection of the MDA-MB-231 cells. When mice were administered zoledronic acid alone, the total tumour burden decreased by 43% compared to placebo treatment. Administration of a combination of zoledronic acid and doxycycline resulted in a 74% decrease in total tumour burden compared to untreated mice. In doxycycline- and zoledronate-treated mice bone formation was significantly enhanced as determined by increased numbers of osteoblasts, osteoid surface and volume, whereas a decrease in bone resorption was also observed. Doxycycline greatly reduced tumour burden and could also compensate for the increased bone resorption. The addition of zoledronate to the regimen further decreased tumour burden, caused an extensive decrease in bone-associated soft tissue tumour burden (93%), and sustained the bone volume, which could result in a smaller fracture risk. Treatment with zoledronic acid in combination with doxycycline may be very beneficial for breast cancer patients at risk for osteolytic bone metastasis.

Bone is one of the most frequent sites for metastasis of breast and prostate cancer. Bone metastases are often associated, especially in breast cancer, with extensive osteolysis of mineralised collagenous bone matrix (Kanis, 1995) and subsequent hypercalcaemia resulting in significant clinical morbidity. Currently, there is no adequate curative regimen for patients with bone metastasis. The existing standard of care for the treatment of breast cancer-related bone metastasis is the bisphosphonate family of drugs that effectively inhibit the cancer-associated resorption. They are also used as therapeutic agents in clinical disorders characterised by increased osteolysis, such as osteoporosis (Berenson *et al*, 2001). Unfortunately, treatment with bisphosphonates is not curative, but randomised control trials have shown that zoledronate, the most potent bisphosphonate, decreases the frequency of skeletal-related events, delays the first occurrence of such an event, and reduces pain in bone-metastatic breast cancer patients (Rosen *et al*, 2004; Kohno *et al*, 2005). During the 2-year daily administration of oral clodronate, one of the earlier bisphosphonates, to patients with operable breast cancer without evidence of metastatic disease the development of bone metastases is significantly reduced (Powles *et al*, 2002). Therefore, preclinical evidence that zoledronate may also be used to prevent the onset of bone metastasis would be very worthwhile.

We have previously shown that doxycycline reduces the tumour burden from breast cancer metastasis in nude mice substantially (Duivenvoorden *et al*, 2002). Doxycycline has several properties relevant for treatment of bone metastasis, as doxycycline can inhibit tumour cell proliferation (Van den Bogert *et al*, 1983; Fife and Sledge, 1995; Duivenvoorden *et al*, 1997) and MMP activity, and accumulates at high concentrations in bone (Golub *et al*, 1991). We have also shown evidence to support the concept that the reduction in tumour burden by doxycycline is likely due to its properties as an inhibitor of tumour cell proliferation and that doxycycline significantly increases several parameters of bone formation (Duivenvoorden *et al*, 2002).

Bisphosphonates are stable pyrophosphate analogues that also accumulate in bone and very effectively inhibit osteoclast-mediated bone resorption. The most potent bisphosphonate is zoledronate, which induces apoptosis of the osteoclast and interferes with the operation of the ruffled border (Green, 2004). In the present study, we combined doxycycline together with zoledronic acid as a possible modality to prevent or reduce the tumour burden from bone metastasis using the experimental breast cancer MDA-MB-231 mouse model.

## MATERIALS AND METHODS

### Cell line

The human breast adenocarcinoma MDA-MB-231 cell line obtained from the American Type Culture Collection (Manassas, VA, USA) was maintained in Dulbecco's minimal essential medium supplemented with 10% fetal bovine serum and antibiotics (100 U ml^−1^ penicillin sodium, 100 *μ*g ml^−1^ streptomycin sulphate and 0. 25 *μ*g ml^−1^ amphotericin B; Invitrogen Canada Inc., Burlington, Ontario, Canada).

### Animals

All protocols for animal studies were reviewed and approved by the Animal Research Ethics Board of McMaster University (Hamilton, Ontario, Canada). Per treatment group 10 female inbred nude (Balb/c nu/nu) mice (Charles River, St. Constant, Quebec, Canada) 5 weeks of age (15–20 g) were used. Control animals, not injected with tumour cells, were also included. Intracardiac injections of MDA-MB-231 cells were done according to Arguello *et al* (1988). Mice were anaesthetised by isoflurane inhalation and the cells (0.1 ml of cell suspension containing 1 × 10^5^ cells) were injected into the left ventricle of the heart using a 26-gauge needle inserted percutaneously near the midline. Twenty-eight days after injection of the cells, high-resolution radiographic scans of all mice in the prone and lateral position were taken under inhaled isoflurane anaesthesia with a Faxitron X-ray system MX-20 (Faxitron X-ray Corporation, Wheeling, IL, USA). The animals were killed and both tibiae, femora, and humeri, and the spinal column were dissected, fixed in formalin, decalcified, using Decalcifier I (Surgipath, Winnipeg, Manitoba, Canada) and embedded in paraffin.

### Treatment

Doxycycline-containing pellets (10 mg per pellet with a timed-release of 21 days; Innovative Research of America, Sarasota, FL, USA) were implanted subcutaneaously 3 days before cancer cell injections. Placebo pellets were used in control animals. At the same time, pellets containing 0.25 mg of 17*β*-estradiol (21 day-release) were implanted in each animal. On the same day, zoledronic acid (Novartis, Dorval, Quebec, Canada) treatment was started, at a dose of 0.2 *μ*g per mouse of zoledronic acid (as 2 *μ*g ml^−1^ PBS), given as a subcutaneous injection. The zoledronic acid injections were repeated nine times, every 2 days.

### Histology and histomorphometry

Tumour burden in the long bones and the spinal column of each mouse were measured using a stereological technique as described before (Duivenvoorden *et al*, 2002). Longitudinal sections (thickness 4 *μ*m) were cut through the middle part of the bone and stained with haematoxylin and eosin (H&E). The total cumulative tumour area per animal was calculated using a point grid (point area of 0.02292 mm^2^) and expressed as bone tumour burden. Histomorphometric measurements were performed on cancellous area of the left and right femora starting 1 mm below the epiphyseal growth plate. Measurements were performed using a digital color camera attached to a light microscope and Northern Eclipse image-analysing software (Empix Imaging Inc., Mississauga, Ontario, Canada). The following parameters were measured: volume occupied by bone, and tumour as a fraction of the total tissue volume (BV/TV and TuV/TV (%), respectively), number of osteoclasts and osteoblasts per length of trabecular bone surface (N.Oc/BS and N.Ob/BS (mm^−1^), respectively), eroded bone surface, active resorption surface beneath or in contact with osteoclasts, and osteoid surface as a fraction of the trabecular bone surface (ES/BS, Oc.S/BS, and OS/BS (%), respectively). To detect tartrate-resistant acid phosphatase (TRAP) activity in the osteoclasts, naphthol AS-BI phosphate (Sigma-Aldrich, Oakville, Ontario, Canada) was used according to van de Wijngaert and Burger (1986).

### Statistical analysis

Tumour burden data were tested for differences between means using Student's *t*-tests. Histomorphometric data were tested for differences between medians using a two-tailed Mann–Whitney test. Differences were considered significant at *P*<0.05.

## RESULTS

Several bisphosphonates have been used in preclinical models of breast cancer bone metastasis. Treatment of mice with ibandronate (Hiraga *et al*, 2001), risedronate (Sasaki *et al*, 1995) or zoledronate (Green *et al*, 2000) results in a reduction of 35, 50 and 80% in osteolytic lesion area as determined by X-ray autoradiography, respectively. Previously, we have shown that doxycycline also significantly reduces tumour burden in the MDA-MB-231 breast cancer bone metastasis model (Duivenvoorden *et al*, 2002). In this study, we describe the effect of zoledronic acid on the skeletal tumour burden in mice concurrently treated with doxycycline.

Doxycycline was administered to Balb/c nu/nu mice as described previously (Duivenvoorden *et al*, 2002), resulting in a delivered dose of approximately 15 mg kg^−1^ day^−1^ starting 3 days before MDA-MB-231 cell injections. Zoledronate was administered at 0.2 *μ*g per mouse every 2 days starting at the same time as the doxycycline treatment. We did not observe any adverse effects of any treatment protocol in the mice over the study period of 28 days. Six long bones (both femora, tibiae and humeri), and each vertebral body of the spinal column of each mouse were screened for the microscopical presence or absence of tumour. Previously, it has been shown that the majority of the tumour burden occurs in these bones (Sasaki *et al*, 1995; Duivenvoorden *et al*, 2002). Occasionally, the maxillae and pelvic bone show presence of tumour.

**Figure 1:**
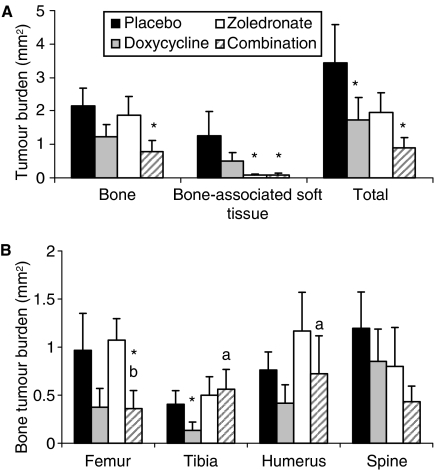
Effect on tumour burden in a mouse bone metastasis model of human breast cancer (MDA-MB-231). Mice received a 21-day timed-release pellets containing 10 mg doxycycline 3 days before intracardiac MDA-MB-231 injections. Other groups of mice received either zoledronic acid (at 0.2 *μ*g per mouse s.c.) every 2 days alone or in combination with doxycycline. A placebo group was also included. Mice were killed 28 days after cell injection. Longitudinal sections of the spine and femora of tumour-bearing animals (*n*=15 in placebo group, *n*=12 in doxycycline-group, *n*=10 in zoledronate-group, *n*=6 in combination group,) were analysed to determine (**A**) tumour burden in bone and bone-associated soft tissue tumour burden and (**B**) average tumour bone burden per evaluated bone (spine, humeri, femora and tibiae). Data represent means±s.e. of the combined values of all animals in each of the groups in at least two separate experiments. ^*^ Significantly different from corresponding placebo group (*P*<0.05). ^a^Significantly different from corresponding doxycycline-alone group (*P*<0.05).^b^Significantly different from corresponding zoledronic acid-alone group (*P*<0.05).

Doxycycline alone and in combination with zoledronate significantly reduced the tumour burden. Figure 1A shows the total tumour burden as determined in the femora and spine. These two bones contributed the most to the total tumour burden per animal (Figure 1B). In the placebo group the total cumulative tumour burden (for spine, femora, tibiae and humeri) per animal amounted to 4.59±1.34 mm^2^ in tumour-bearing animals (*n*=15), whereas the total tumour burden in the doxycycline group (*n*=12) was 2.28±0.83 mm^2^, a significant reduction of 50%. In the group of animals that received zoledronate alone (*n*=10) the tumour burden was 3.62±1.05 mm^2^, a number that decreased to 2.16±0.74 mm^2^ when doxycycline was added to the treatment protocol (*n*=6). The tumour burden in the latter group was also significantly decreased when compared to the placebo-treated animals. Interestingly, zoledronic acid effected primarily a reduction in bone-associated soft tissue tumour burden, whereas doxycycline decreased both the bone- and associated soft-tissue tumour burden. Zoledronate caused a 93% reduction in soft-tissue tumour burden, irrespective of doxycycline treatment, whereas the total tumour burden was merely reduced by 74 and 43%, in the presence or absence of doxycycline, respectively. The combination treatment decreased the tumour burden of both bone and associated soft tissue to a larger extent than either single treatment alone (Figure 1A), although this trend did not reach statistical significance. The numbers of mice per group ended up dissimilar, even though, per repeated experiment, five mice per group were injected. The numbers also excluded mice that died very soon after tumour cell injection and ones that did not develop tumours. Moreover, the control and doxycycline-treated mice included mice from previous experiments (Duivenvoorden *et al*, 2002).

**Figure 2:**
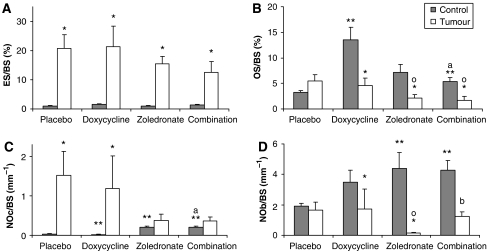
Effect of doxycycline treatment on bone histomorphometric parameters in a mouse bone metastasis model of human breast cancer (MDA-MB-231). Longitudinal sections of the femora were analysed to determine (**A**) eroded bone surface as a fraction of the trabecular bone surface (%), and (**B**) osteoid surface as a fraction of the trabecular bone surface (%), (**C**) number of osteoclasts per mm trabecular bone surface, and (**D**) number of osteoblasts per mm of trabecular bone surface. Data represent means±s.e. of the combined values of all animals in each of the groups in at least two separate experiments (*n*⩾3). ^*^ Significantly different from corresponding control group (no tumour cells injected) (*P*<0.05). ^**^ Significantly different from placebo control animals (no tumour cells injected) (*P*<0.05). ^o^Significantly different from placebo tumour-bearing animals (*P*<0.05). ^a^Significantly different from corresponding doxycycline-alone group (*P*<0.05). ^b^Significantly different from corresponding zoledronic acid-alone group (*P*<0.05).

Bone histomorphometry showed that both doxycycline and zoledronate significantly increased several parameters of bone formation in the femora, including osteoid volume (data not shown), osteoid surface and the number of osteoblasts per bone surface (see Table 1, Figure 2B and D). When zoledronic acid alone was used, there was a dramatic and significant increase in bone volume in control animals, which was maintained in the tumour-bearing animals (Table 1). The addition of doxycycline to the regimen of zoledronic acid sustained the increase in bone volume and also induced a concomitant decrease in tumour volume even though the difference did not reach significance.

**Figure 3:**
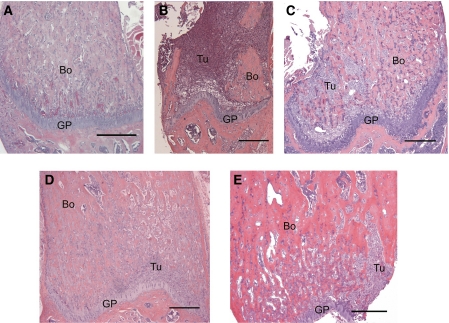
(**A**) Histological appearance of the left femur of a control mouse (untreated, no cell injections) stained with H&E. (**B–E**) Histological appearance of skeletal tumours in the left femora of mice injected with MDA-MB-231 cells after H&E staining. Mice were treated with (**B**) placebo, (**C**) doxycycline, (**D**) zoledronic acid, or (**E**) with both doxycycline and zoledronic acid. (Original magnification × 100, Bar = 150 *μ*m). Bo, bone; GP, growth plate; Tu, tumour.

**Figure 4:**
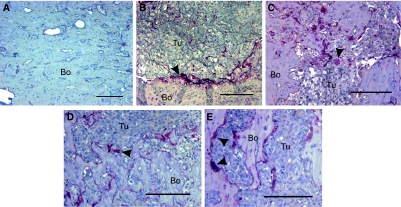
(**A**) Histological appearance of the left femur of a control mouse (untreated, no cell injections) stained for TRAP. No osteoclasts were observed. (**B**–**E**) Histological appearance of skeletal tumours in the left femora of mice injected with MDA-MB-231 cells showing TRAP-positive osteoclasts (arrowheads). Mice were treated with (**B**) placebo, (**C)** doxycycline, (**D**) zoledronic acid or (**E**) with both doxycycline and zoledronic acid. (Original magnification × 400, Bar=20 *μ*m). Bo, bone; Tu, tumour; arrowheads, osteoclasts.

The presence of breast cancer has a profound effect on bone. Skeletal MDA-MB-231 tumours showed extensive marrow colonisation, significant osteolysis, and cortical perforation. Whereas in control animals, hardly any osteoclasts could be found, the number of osteoclasts and the eroded bone surface increased markedly in tumour-bearing bones (Figure 2A and C). In contrast, the structure of the trabecular bone in mice treated with zoledronic acid alone and in combination with doxycycline (Figure 3D and E) seemed better preserved, even in the presence of tumour cells. The mice treated with doxycycline in the presence or absence of zoledronic acid both showed a significant increase in the osteoid surface (Figure 2B); however, this effect could not be maintained in the presence of tumour. Haematoxylin and eosin sections of the femur (Figure 3) clearly show the osteolysis induced by the presence of tumour. Sections stained for TRAP show the increased presence of osteoclastic activity at the tumour–bone interface (Figure 4). In the presence of tumour, we observed noticeable osteolysis on H&E-stained sections and increased osteoclastic activity at the tumour–bone interface after TRAP staining. We also noticed less TRAP-positive osteoclasts present on the bone surface in both zoledronic acid groups, in the presence or absence of doxycycline (Figure 2C).

A major feature of breast cancer bone metastasis is the uncoupling of bone remodeling. We also observed this phenomenon in tumour-bearing bones and show that doxycycline and zoledronic acid can improve this to some level by increasing bone formation. In doxycycline-treated tumour-bearing mice, the tissue volume occupied by bone was significantly increased, 29.1% compared to 22.7% in placebo (Table 1). The bone volume further increased to 32.8 and 38.7% in animals treated with zoledronic acid alone and in combination with doxycycline, respectively. These represented levels of bone volume that were higher than in control mice. This was accompanied by an increase in bone formation parameters (number of osteoblasts (Figure 2D) and osteoid surface (Figure 2B) and volume (Table 1)) and a concomitant decrease in bone resorption parameters, such as number of osteoclasts (Figure 2C) and eroded bone surface (Figure 2A). Interestingly, in control mice, zoledronic acid alone resulted in an increase in the number of both osteoclasts and osteoblasts, suggesting an overall increase in bone remodeling, even in the absence of tumour cells. Moreover, in autoradiographs of mice treated with zoledronate, we also observed dense bone nodules at the sites of rib articulation (data not shown). The aberrant bone formation was noted in zoledronate-treated animals irrespective of doxycycline treatment or the presence of tumour cells.

## DISCUSSION

Using the same bone metastasis model, zoledronate has been shown to markedly reduce the area of osteolytic lesions as detected by X-ray autoradiography, independent of time of administration (concurrently with the tumour cell injections or after the establishment of bone metastasis) (Peyruchaud *et al*, 2001) and of the dose administered (0.2, 1 and 5 *μ*g per mouse; Green *et al*, 2000). When GFP-labelled MDA-MB-231 cells are used, the effect of zoledronate on the fluorescence as a measure of the amount of tumour cells is less pronounced than on the osteolytic lesion area (Peyruchaud *et al*, 2001). Similarly, treatment with olpadronate, another bisphosphonate, demonstrates that the tumour burden in the initial phases is more heavily impacted and returns to vehicle-treated levels by the end of the 47-day treatment. During the same time frame, radiography shows a sustained and extensive decrease in osteolytic lesions (van der Pluijm *et al*, 2005), thus substantiating the primary mechanism of action of bisphosphonates as anti-resorptive. We observed primarily a large reduction in bone-associated soft tissue tumour burden (93%) after administration of zoledronate. This is also supported by data using a 4T1/luc mouse breast cancer model showing that zoledronic acid results in an inhibition of visceral metastasis (Hiraga *et al*, 2004) and by Corey *et al* (2003), who provided evidence to suggest that the inhibitory effects of zoledronate may not only be associated to its effect on osteolysis, but also on cancer cell proliferation and apoptosis.

When comparing both compounds, our results showed that doxycycline induced a larger decrease in tumour burden, whereas zoledronate was more capable of sustaining the bone volume, in spite of an increase in bone resorption. Similar results were reported using osteoblastic LuCaP23.1 or osteolytic PC-3 prostate cancer cells injected into mouse tibiae. Zoledronic acid treatment also induces a decrease in tumour volume and a concomitant increase in bone volume (Corey *et al*, 2003). When we administered both drugs the result was a substantial decrease in tumour burden and a sustained bone volume. Future investigations will focus on potential effects on local concentrations of each of the osteotropic drugs in the bone and on the specific targets of both drugs. The main target of zoledronic acid appears to be the induction of apoptosis (Corey *et al*, 2003; Quinn *et al*, 2005), whereas doxycycline induces a G1 cell cycle arrest (Van den Bogert *et al*, 1986). Hiraga *et al* (2001) describe a substantial increase in apoptotic MDA-MB-231 cells in the bones of mice treated with ibandronate, especially at the bone–tumour interface. Several other mechanisms of action have also been suggested for both drugs. *In vitro*, zoledronate has also been reported to stimulate osteoprotegerin production in primary human osteoblasts (Viereck *et al*, 2002), inhibit prostate cancer cell proliferation (Corey *et al*, 2003), MMP activity (Boissier *et al*, 2000) and adhesion to bone extracellular matrix (Boissier *et al*, 1997).

Whereas the current study clearly demonstrates the benefit of combined treatment when both drugs are administered simultaneously starting 3 days before cell injections, future experiments will include sequential administration, and starting several weeks after the cell injections. This will help to determine whether doxycycline could also be beneficial for patients with already established bone metastasis and who are currently receiving zoledronic acid and vice versa. In conclusion, doxycycline greatly reduced tumour burden and could also compensate for the increased bone resorption frequently associated with bone metastasis from breast cancer. The addition of zoledronate to the treatment regimen further decreased tumour burden and effected an extensive decrease in bone-associated soft tissue tumour burden, independent of doxycycline treatment. Moreover, the combination also sustained bone volume, which could greatly reduce the risk of fracture. Treatment with zoledronic acid in combination with doxycycline may be very beneficial for breast cancer patients at risk for osteolytic bone metastasis.

## Figures and Tables

**Table 1:** Effect of treatment with doxycycline or zoledronate alone or combined on bone and tumour volume in mouse bone metastasis model of human breast cancer (MDA-MB-231) as measured by bone histomorphometry on longitudinal sections of the femora

	**Bone volume BV/TV (%)**			
	**Placebo**	**Doxycycline**	**Zoledronic acid**	**Doxycycline and zoledronic acid**
Control animals	29.5±0.32	29.2±0.97**	37.0±1.79**	37.6±1.84**^a^
Tumour-bearing animals	22.7±1.40*	29.1±2.69**	32.8±1.03**	38.7±5.15
				
	**Tumour volume TuV/TV (%)**			
Tumour-bearing animals	31.6±3.68	26.4±6.38	28.3±3.64	15.0±4.97^a^

Data represent means±s.e. of the combined values of all animals in each of the groups in at least two separate experiments.

* Significantly different from corresponding control (no tumour) group (*P*<0.05).

** Significantly different from corresponding placebo group (*P*<0.05).

a Significantly different from corresponding doxycycline-alone group (*P*<0.05).
